# Adjuvant Lineage-Negative Cell Therapy as a Potential Silencer of the Complement-Mediated Immune System in ALS Patients

**DOI:** 10.3390/jcm10225251

**Published:** 2021-11-11

**Authors:** Anna Sobuś, Bartłomiej Baumert, Monika Gołąb-Janowska, Piotr Kulig, Edyta Paczkowska, Karolina Łuczkowska, Dorota Rogińska, Alicja Zawiślak, Sławomir Milczarek, Bogumiła Osękowska, Wioletta Pawlukowska, Agnieszka Meller, Karolina Machowska-Sempruch, Agnieszka Wełnicka, Przemysław Nowacki, Bogusław Machaliński

**Affiliations:** 1 Department of General Pathology, Pomeranian Medical University, 70-111 Szczecin, Poland; ania.sobus@gmail.com (A.S.); bbaumert@pum.edu.pl (B.B.); piotrkulig@interia.eu (P.K.); edyta.paczkowska@pum.edu.pl (E.P.); karolinaluczkowska58@gmail.com (K.Ł.); doroginska@gmail.com (D.R.); alicja.zawislak@pum.edu.pl (A.Z.); slawek.milczarek@gmail.com (S.M.); bogumilaosekowska@gmail.com (B.O.); 2 Department of Neurology, Pomeranian Medical University, 71-252 Szczecin, Poland; monikagj@op.pl (M.G.-J.); wpawluko@pum.edu.pl (W.P.); agoschorska@gmail.com (A.M.); karolinamachowska88@gmail.com (K.M.-S.); awelnicka@gmail.com (A.W.); przemyslaw.nowacki@pum.edu.pl (P.N.); 3 Department of Medical Rehabilitation and Clinical Physiotherapy, Pomeranian Medical University, 71-210 Szczecin, Poland

**Keywords:** amyotrophic lateral sclerosis, lineage-negative cells, cell therapy, innate immunity, complement system

## Abstract

ALS remains a fatal, neurodegenerative motor neuron disease. Numerous studies seem to confirm that innate immune system is involved in the pathophysiology of ALS. Hence, the assessment of the complement system and attempts to modify its activity remain the target of medical intervention in ALS. In the present study, three intrathecal administrations of autologous bone marrow-derived lineage-negative (Lin^–^) cells were performed every 6 weeks in 20 sporadic ALS patients. The concentrations of various complement components in the cerebrospinal fluid and plasma at different time points after cell injection were quantified using a Luminex multiplex. The results of the complement system were correlated with the level of leukocytes, neutrophils, lymphocytes, fibrinogen and CRP in the peripheral blood and the functional status of ALS patients using Norris and ALS-FRSr scales. The study showed a statistically significant decrease in plasma C3b concentration in all 7th days after cell application. In parallel, a peak decrease in neutrophil count and CRP level was observed on days 5–7, with a simultaneous maximum clinical improvement on days 7–28 of each Lin^–^ cell administration. Adjuvant Lin^–^ cell therapy appears to have the silencing potential on the complement-mediated immune system and thus suppress pro-inflammatory reactions responsible for neurodegeneration. However, further in-depth studies are necessary to address this issue.

## 1. Introduction

Amyotrophic lateral sclerosis (ALS) is a lethal, progressive disease specifically affecting both upper motor neurons (UMN) and lower motor neurons (LMN). The clinical characteristic of ALS is a combination of UMN and LMN signs and symptoms as well as features of frontotemporal dementia [1]. Approximately 5–10% of cases are familial and 90–95% sporadic [2]. Albeit the etiology of ALS is still not entirely elucidated, it is suggested that multiple mechanisms such as mitochondrial dysfunction [3], neuroinflammation [4], oxidative stress [5] and protein aggregation [6] are embroiled in its pathogenesis. There is an increasing number of evidence that immune system, with emphasis on innate immunity, plays a particularly important role. In the animal study, overexpression of several proinflammatory cytokines such as TNF-α, IFN-γ, IL-2, IL-6, IL-10 and CCL3 was detected in hSOD1G93A mice. Moreover, what is noteworthy, the same study depicts the involvement of macrophages, monocytes and mast cells [7]. Another study conducted by Sta et al. demonstrated the presence of activated CD3^+^ and CD8^+^ T-cells in the spinal cord and motor cortex of ALS patients. On top of that, activation of complement system via the classical pathway was illustrated [8]. Complement is an important part of the innate immune system and can be activated in a cascade of enzymatic reactions via the classical, alternative and lectin pathway. All of the abovementioned ways converge in a terminal pathway which eventually forms the membrane attack complex (MAC) [9]. Complement activation appears to be an early event in ALS pathophysiology. Bahia El Idrissi et al. studied complement activation on motor end-plates in ALS patients. C1q and MAC were detected in motor end-plates in post-mortem ALS donors whereas not present in a control group. In addition, the complement C3/C3b was found in the intercostal muscle of ALS donors at the motor nerve terminal and terminal Schwann cells but not in controls. Moreover, herein stated, MAC depositions were present on innervated motor end-plates suggesting that complement activation might precede denervation. Complement regulatory proteins CD55 and CD59 were also deposited on effected motor end-plates suggesting an attempt to control the process, yet this response seems not to be effective [10]. In another research, levels of selected markers of the lectin complement pathway as well as complement activation products in the peripheral blood and cerebrospinal fluid (CSF) were measured. It has been suggested that the terminal part of the complement system is activated in ALS patients in a non-canonical manner. In addition, the novel finding in this study is that plasma levels of collectin-11 and C4 were significantly increased [11]. In another animal study, C3 activation products i.e., C3b/iC3b were detected on neurons and in neuromuscular junction in hSOD1G93A mice during the pre-symptomatic stage [12]. Lee et al. showed in hSOD1G93A mice increased immunolabelling for C3b in the lumbar spinal cords. Moreover, C3b deposition appeared primarily on motor neurons and microglia [13].

Abovementioned studies suggest that innate immunity and particularly complement system play an important role in ALS pathophysiology, presumably on an early stage of the disease. Although further research is needed, inhibiting complement system might be a potential therapeutic approach [14,15].

Nowadays many research teams are looking for optimal adjuvant cell therapy protocols to modulate the immune system and thus prevent neurodegeneration. We also thoroughly studied ALS pathophysiology with regards to immunological response and clinical outcomes. In a preliminary study [16] we investigated whether autologous bone marrow-derived Lin^–^ cells intrathecal administration is safe and feasible in ALS patients and assessed changes in concentration of several growth and pro-inflammatory factors and miRNA in plasma and CSF. We have focused particularly on the population of Lin^–^ cells as we have previously proved that, under stress conditions, they express neurotrophic factors more efficiently than other nucleated cells obtained from bone marrow [17]. In the subsequent study, we further evaluated correlations between patients’ clinical outcome and concentrations of various neurotrophins and several miRNA expression profiles [18]. In recent study, we assessed effect of repeated intrathecal Lin^–^ cell administration on the regulation of some immunological pathways [19].

The aim of the present study is to further investigate the impact of multiple intrathecal Lin^–^ cell administration on immune response with emphasis on innate immunity and particularly the complement system.

## 2. Materials and Methods

### 2.1. Patients

The presented study was part of a prospective, non-randomized, single center clinical trial (trial number NCT02193893) for subjects with sporadic form of ALS. The study was accepted by a local ethics committee of Pomeranian Medical University in Szczecin and conducted according to the Declaration of Helsinki. As this preliminary experiment is still a safety and proof of concept study, no placebo control group was intentionally planned. Altogether, 20 patients with ALS, aged between 27 and 62 (mean age 54.7 ± 7.7 years) were included in the study. The inclusion criteria were as follows: (i) age between 18 and 65 years; (ii) ability to express informed consent; (iii) probable or certain sporadic ALS diagnosis (based on the El Escorial Revised Criteria); (iv) no family history of ALS; (v) 3-month observation and Riluzole treatment prior to the cells administration; (vi) forced vital capacity greater than 50%; (vii) exclusion of comorbidities (cancer, diabetes, cardiovascular disease, chronic kidney and liver disease) and patients receiving drugs which might influence bone marrow function.

### 2.2. Neurological Assessment

The progression of the disease was assessed using two functional scales: Norris scale and ALS Functional Rating Scale Revised (ALS-FRSr). Both assessment scales were used before each cell administration as well as 3, 5, 7, 28 days after each procedure and 3 months after the last injection. 

### 2.3. Lineage-Negative Cells Isolation and Administration

Bone marrow Lin^–^ cells were administered three times at 6-week intervals. The bone marrow (BM) was collected three times from the posterior iliac crest on day 0 of each procedure in local anesthesia by a qualified hematologist. Afterwards, BM was subjected to density gradient centrifuging to obtain mononuclear cells (MNC) which were further processed to negative isolation using immunomagnetic method with a commercially available kit (Miltenyi Biotec, Auburn, AL, USA) following the manufacturer’s protocol. All the obtained cells were subsequently administered to the subarachnoid space via the lumbar puncture. As reported in our previous papers we have not observed any adverse effects of the Lin^–^ cells administration (16–18). 

### 2.4. Multiplex Analysis of Complement Components Concentration in Plasma and CSF

The concentrations of selected complement components were assessed in plasma and cerebrospinal fluid (CSF) collected before cells administration and one week after each of the three injections. Both sample types were centrifuged to remove the cellular content and stored in 500 µL aliquots in −80 °C upon analysis. To assess the concentrations of selected complement components (C1q, C3b, C4, complement factor B and complement factor H) in plasma and CSF a multiplex fluorescent bead-based immunoassays were performed according to manufacturer’s instructions (Luminex Corporation, Austin, TX, USA). In each samples the concentrations were measured in two replicates and the final concentrations were calculated based on the average readings in relation to 6-point standard curves. 

### 2.5. Blood Examinations

The blood tests were performed on the day of cell application, 1, 3, 5, 7 and 28 days after each cell injection. To evaluate the intensity of the ongoing systemic immune reactions, the count of leukocytes, neutrophils and lymphocytes was assessed and the concentrations of C-reactive protein (CRP) and fibrinogen were measured.

### 2.6. Statistical Analysis 

All statistical calculations were performed using STATICTICA 13 software. Wilcoxon signed rank test was used to find the differences of analyzed parameters in certain timepoints in comparison to day 0 of each procedure. The correlations between assessed parameters were calculated using Spearman’s correlation rank test.

## 3. Results

The overall characteristic of the study group presented in Table 1. There were no statistically significant differences in the number of administered Lin^–^ cells between the consecutive injections.

**Figure 1:**
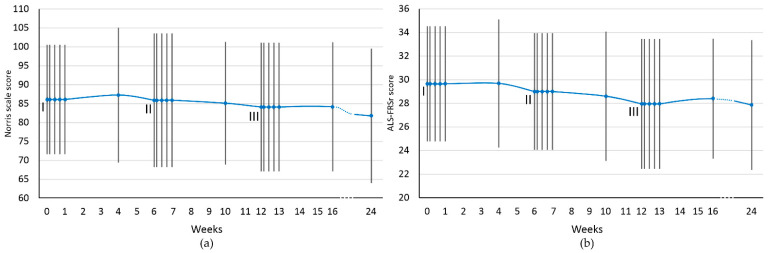
Functional scales assessment scores for the whole study group. (**a**) Norris scale; (**b**) ALS-FRSr scale. I, II, III—cell administration procedure. The results are presented as the mean values. Blue dots represent the analyzed timepoints. Vertical lines represent SD.

The results of initial assessment with functional scales were 86.0 ± 14.45 using Norris scale and 29.65 ± 4.88 for ALS-FRSr. Mean scores obtained in consecutive assessment timepoints are presented as trends in Figure 1.

In general, as a result of the applied cell therapy, stabilization or improvement of the functional state was observed each time reaching a maximum from the 7th to the 28th day after cell administration. Thereafter, a gradual clinical deterioration was observed at weeks 4–6 between cell application. With cessation of cell therapy, there was a clear downward trend in both functional scales (from 4 to 12 weeks after the third administration).

### 3.1. Complement Components Concentrations

**Figure 2:**
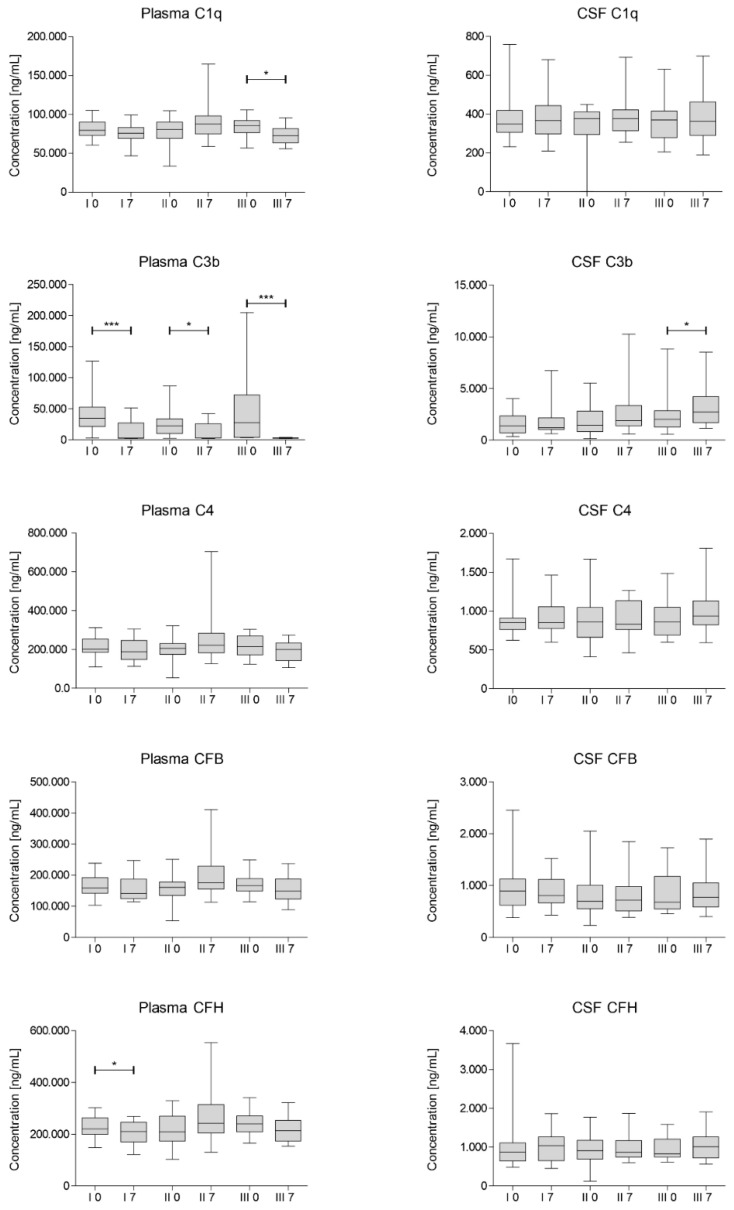
Multiplex Luminex median concentration of selected complement components assessed for each cell administration (I, II, III) on day 0 and 7. CSF—cerebrospinal fluid; CFH—complement factor H; CFB—complement factor H. Significance of the changes between analyzed timepoints in comparison to day 0 of each administration was assessed using Wilcoxon signed-rank test. Level of significance—* *p* < 0.05, *** *p* < 0.001.

The concentrations of C1q, C3b, C4, complement factor B (CFB) and H (CFH) were assessed in blood plasma and CSF in samples collected on day 0 and 7 after each of the three injections of Lin^–^ cells. The results of the analysis are presented in Figure 2.

We have observed a statistically significant decrease in C3b concentrations on day 7 after each of the cell administrations in plasma. Moreover, we have also noted a significant decrease in CFH concentration after first injection of Lin^–^ cells and in concentration of C1q in plasma after the last procedure. On the other hand, the average concentration of complement factors increased in CSF on day 7 after each injection, however mostly statistically insignificant. It is worth noting that in general the concentrations of complement components in CSF are at least 10-fold lower than in plasma and differ more between analyzed samples.

### 3.2. Blood Cell Count Analysis

**Figure 3:**
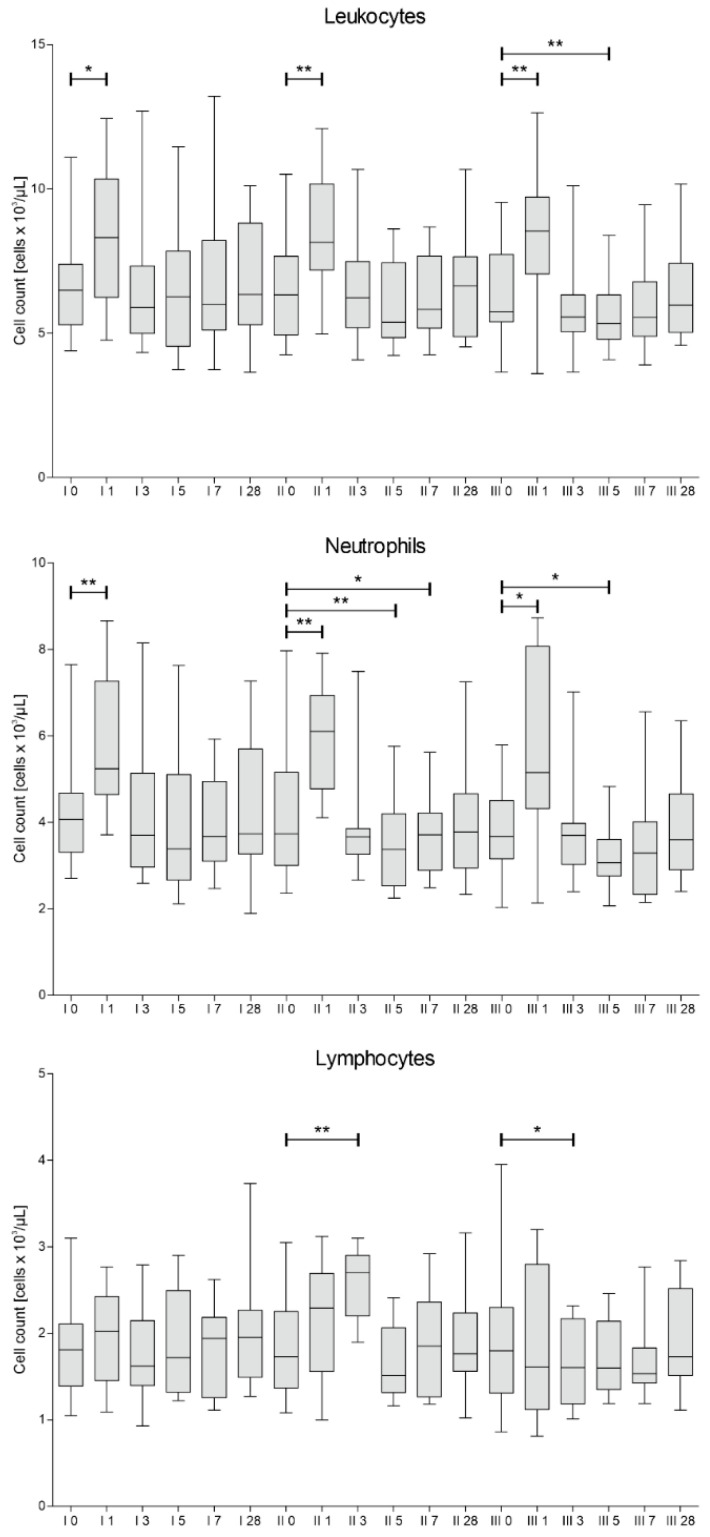
Median white blood cells count with differential into neutrophils and lymphocytes measured on days 0, 1, 3, 5, 7 and 28 for each of three cell administration procedures (I, II, III). Significance of the changes between analyzed timepoints in comparison to day 0 of each administration was assessed using Wilcoxon signed-rank test. Level of significance—* *p* < 0.05, ** *p* < 0.001.

The count of white blood cells, neutrophils and lymphocytes was assessed in blood samples collected from patients on the day of each cell injection, 1, 3, 5, 7 and 28 days after each procedure. The results of this analysis are presented in Figure 3. 

It is noteworthy, that after each administration of the cells, we have observed a rapid (on day 1) increase of leukocytes and neutrophils, probably due to invasive therapeutic intervention. Afterwards, the level of leukocytes and neutrophils decreased, reaching its minimum on days 5–7.

### 3.3. Plasma Fibrinogen and C-Reactive Protein Concentrations

**Figure 4:**
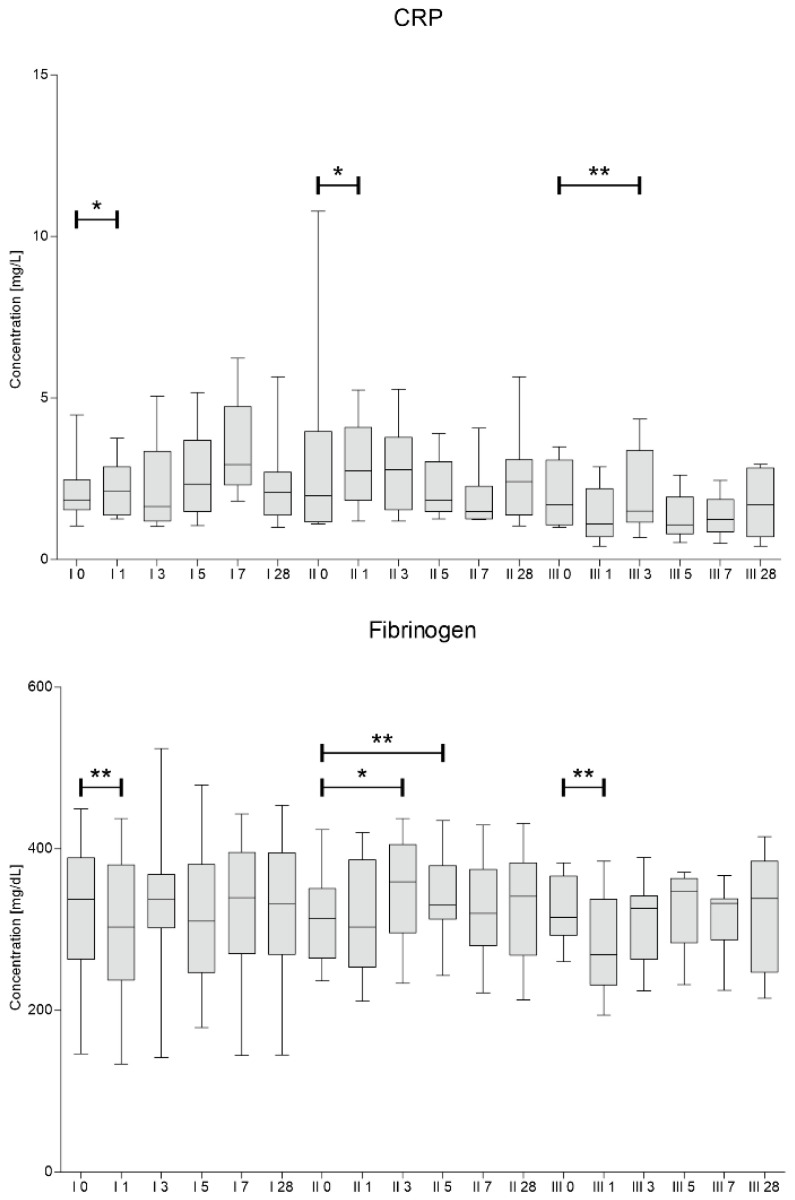
C-reactive protein and fibrinogen median concentrations assessed on days 0, 1, 3, 5, 7 and 28 for each of three cells administration procedures (I, II, III). Significance of the changes between analyzed timepoints in comparison to day 0 of each administration was assessed using Wilcoxon signed-rank test. Level of significance—* *p* < 0.05, ** *p* < 0.001.

Concentrations of fibrinogen and CRP were assessed in blood samples collected at the same time points as for the analysis of blood counts. The results are presented in Figure 4. 

We have observed a significant decrease in fibrinogen concentration one day after the procedure of first and third Lin^–^ cells injection. In contrary, the concentrations of CRP increased on day 1 during first and second procedure. Then, a downward trend in CRP was visible on days 5–7 after second and third cell administration.

### 3.4. Correlations

Correlations between analyzed parameters from plasma and CSF were calculated using Spearman’s rank correlation test. Table 2 presents only significant correlations of selected factors. Concerning age, a very strong negative correlation with the plasma level of C3b (3rd administration, day 0) and a positive correlation with C4 in CSF (3rd administration, day 0) have been demonstrated. Interestingly, the only factor positively correlated with disease duration was the plasma concentration of C3b before treatment initiation. The concentration of only two elements of the complement system in plasma and CSF positively correlated with each other, C3b and C4, respectively. 

## 4. Discussion

ALS is a fatal, progressive condition of not entirely elucidated pathogenesis, yet it is well-established that several pro-inflammatory mechanisms contribute to development and it is speculated that to progression as well [20,21]. There are various possible immune pathways considered, however, still relatively little is known about complement and its role in ALS. Complement system plays particularly important role in innate immune response via promoting inflammation (C5a acts as a chemoattractant for neutrophiles [22]), opsonization and thereby enhancing phagocytosis. In addition, MAC causes pathogens’ cells lysis. Moreover, it is appreciated that complement acts as a bridge which binds together innate and adaptive immunity [23]. 

Since it is well-established that immune system significantly contributes to the development of ALS, modulating of immune response could be a promising therapeutic strategy. Stem cell therapy was initially proposed to replenish continuously lost motor neurons as stem cells possess the ability to differentiate into any or limited cell type [24]. Alterations in neurotrophins and their receptors have been found in ALS [25]. Although administration of BDNF did not show the improvement of patients’ outcome, a not statistically significant improvement trend was noticed [26,27]. Another approach is to transplant cells that have the ability to constantly secrete neurotrophic factor, and thus enhance the neuroprotective effect. In the randomized, double-blind, placebo-controlled phase 2 trial autologous bone marrow-derived mesenchymal stem cells (MSCs), induced to secrete neurotrophic factors (NTFs) were administered to the participants. Results supported proposed paracrine mechanism of action of MSC-NTFs and the combined effects on both neuroprotection and neuroinflammation. On top of that, significant efficacy in a prespecified subpopulation of rapid progressors was demonstrated [28].

Autologous lineage-negative stem cell transplantation in ALS patients has been thoroughly investigated by our research team for past several years. Initially, we established that this procedure is both safe and feasible [16]. In subsequent studies, we demonstrated that beneficial effect of autologous Lin^–^ stem cell transplantation was predominantly due to secretion of neurotrophins and angiogenic factors [18,29]. Eventually, we repeated the procedure three times in ALS patients showing that triple administration is safe and effective. Obtained results suggest that procedure’s clinical effect is not only due to the paracrine secretion of neurotrophins, but also because of the modulation of immunological pathways [19]. Moreover, we depicted that ALS patients improve in articulatory functions after repeated autologous Lin^–^ stem cell transplantation [30]. It should be noted that the number of administered Lin– cells differed between patients in presented study. Each time all the cells obtained via immunomagnetic isolation were injected intrathecally. However, the statistical analysis did not reveal any statistically significant correlation between the number of Lin– cells applied and the clinical outcome nor the levels of assessed proteins. Another interesting observation is that the influence of cell injection on functional state was most pronounced after the first administration procedure. This may suggest that the first procedure has the strongest adjuvant effect, while the consecutive ones only sustain it. Moreover, in all the studies mentioned above, as well as in the current study we have noticed that some patients tend to respond favorably to the treatment regardless of the number of cells administered, while others have not seen any beneficial effects of the procedure. The observation made seems to confirm that the response to adjuvant cell therapy is complex and multifactorial and still remains not entirely elucidated.

Since ALS etiology is at least partially inflammatory, it can be assumed that complement system is involved in diseases’ development and progression. Apostolski et al. have reported that C4 concentrations are elevated in the serum of ALS patients in comparison to healthy volunteer group [31]. C5a is proved to be a chemoattractant for neutrophiles [22] and therefore might affect the WBC number. The post-mortem studies of central nervous system material from ALS patients have also revealed the increased deposition of the complement molecules, in particular C3 in the site of degenerating neurons [32]. Complement activation seems to be an early event in ALS pathophysiology since MAC depositions were present on innervated motor end-plates. C1q and MAC were detected in motor end-plates in post-mortem ALS donors. As has already been stated above, the complement C3/C3b was found in the intercostal muscle at the motor nerve terminal and terminal Schwann cells [10]. In the animal study dysregulation of the complement system at pre-symptomatic stage was shown [13]. Although the role of complement in ALS pathophysiology becomes more apparent, still there is a shortage of research in this particular field. Many studies were conducted solely on animal models. Therefore, we intended to fill this gap. What is noteworthy, to our best knowledge, the current study is the first to focus on complement after repeated administration of autologous Lin^–^ stem cells. We aimed to evaluate if this approach can modulate the disease and perhaps prevent or delay neurodegeneration.

In order to assess the disease progression two functional scales (Norris scale and ALS-FRSr) were applied. Repeated administration of Lin^–^ stem cells resulted in transient either neurological improvement or stabilization in both of used scales, especially from day 7 to 28 of each procedure. Between cell therapies (from 4th to 6th week) gradual deterioration was noticed. After the third administration, there was a clear negative trend in both functional scales (from 4th to 12th weeks). Due to the lack of a control group in the presented study, it is difficult to assess if the pace of ALS-FRSr scores deterioration resulted from the applied cell therapy or the natural course of the disease. Especially that the results based on different cohorts of patients with ALS show large discrepancies in the slope of deterioration measured using functional scales between patients [33]. It might be hypothesized that immunomodulatory and neurotrophic effects are not permanent and maintaining clinical effect may require repetitive stem cell boosts.

The concentrations of C1q, C3b, C4, complement factor B (CFB) and H (CFH) were assessed in blood plasma and CSF in samples collected on day 0 and 7 after each of the three injections of Lin^–^ cells. Our paramount finding is statistically significant decrease in concentration of C3b in plasma 7 days after each cell administration. There are number of studies showing that C3b shifts the metabolic profiles of various leukocytes to support pro-inflammatory reactions [34]. The decrease of concentration of C3b on every 7th day in plasma interestingly correlated with a peak decrease in neutrophil count observed on days 5–7. Moreover, baseline neutrophil levels prior to 2nd and 3rd administration correlated positively with C3b and C1q concentration in plasma respectively. Parallel, a peak decrease in CRP level on days 5–7 after second and third Lin^–^ cell administration was confirmed. At the same time, baseline CRP level positively correlated with the plasma concentration of C3b and C4. This laboratory findings are consistent with a maximum clinical improvement from day 7 of each Lin^–^ cell administration. Obtained results strongly suggest that neurotrophic and immunomodulatory effect of stem cells affect not only innate, but also adaptive immunity as complement binds them together [23]. It is worth to note that concentration of C1q in plasma also significantly decreased 7 days post the third Lin^–^ cell administration. Studies in the SOD1(G93A) mouse suggest that activation of complement via C1q and C3b deposition at the motor end-plate precedes neurodegeneration [10]. Hence, reducing complement-induced inflammation could be an important therapeutic strategy to treat ALS [13]. Alterations in complement proteins in CSF were predominantly statistically insignificant. However, the concentration of C3b and C4 in CSF and plasma positively correlated with each other. Similarly, the baseline CRP level before first and third administration were positively correlated with the concentration of C1q in CSF. In our previous research [16,18] we established that peak of the neurotrophin secretion is during first 3–5 days after Lin^–^ cells application. Therefore, collecting CSF sample after seven days is presumably too late. In order to obtain informative results CSF should be collected several times during the first days after the procedure. However, lumbar puncture is an invasive procedure and we decided to perform it twice, before and after seven days of each Lin^–^ cell injection due to ethical reasons. Broadly speaking, we showed no difference between complement proteins after seven days in CSF. It might be due to the fact that local response i.e., in CSF occurs quicker than systemic one. Moreover, systemic response lasts longer and hence clinical improvement measured using functional scales is visible approximately up to 28 days after the procedure. Several times lower concentrations of complement factors in CSF compared to plasma also draw attention. 

Autologous Lin^–^ stem cell application modulates whole immune system via multiple mechanisms and therefore interacts with the complement system in many ways. There are multiple other factors that may interact with complement system and hence can alter concentrations of complement proteins. For example, pentraxins like CRP or serum amyloid protein P have a dual relationship with the complement system. Initially, pentraxins activate complement by binding its first component C1q. However, the emerging inflammation needs to be limited to the target area. Therefore, pentraxins inhibit complement at the C3b stage to prevent excessive damage [35]. 

It was previously confirmed that the complement plays a key role in the pathogenesis of ALS at an early, pre-symptomatic stage [10]. It needs to be highlighted that in our study, plasma concentration of C3b prior to the therapy was the only factor positively correlated with disease duration. At the same time, Lin^–^ cell therapy had a suppressive effect on the C3b and C1q elements in the plasma. In further research trials, a group of patients with a short disease duration should be recruited, who could potentially benefit the most from the therapy by preventing neurodegeneration.

In conclusion, adjuvant Lin^–^ cell therapy appears to have the silencing potential on the complement-mediated immune system and thus might suppress pro-inflammatory reactions responsible for neurodegeneration. However, further in-depth studies are necessary to address this issue.

### Study Limitations

While we put effort into the design of our study, we encountered several limitations. First of all, there was no placebo-controlled study planned at this stage of the experiment. We have focused mainly on safety, feasibility and pathophysiological evaluation of the immune processes taking place in patients with ALS during cell therapy. Subsequently, CSF was collected only twice i.e., on the day of the procedure and after 7 days what also was meant to diminish the number of invasive procedures that participants were to undergo. Moreover, our observation time was relatively short since patients were from remote parts of the country and therefore were not able to regularly and often visit our center, especially since the patients’ condition kept deteriorating. Despite the above, we believe that our study still provides new insights into the mechanisms of complement-mediated immune system in ALS patients.

## Figures and Tables

**Table 1:** Characteristic of study group.

Age (Mean ± SD, Years)		54.7 ± 7.68
Gender (male/female)		11/9
Disease duration (mean ± SD, months)		31.3 ± 26.68
Disease onset (bulbar/limb)		5/15
Number of administered Lin^–^ cells (mean ± SD)	**1st administration**	5.5 × 10^6^ ± 4.4
	**2nd administration**	8.9 × 10^6^ ± 8.1
	**3rd administration**	6.4 × 10^6^ ± 4.2
ALS-FRSr score vs. baseline score (mean values for all patients ± SD)	**28 days post** **1st administration**	+0.03 ± 1.29
	**28 days post** **2nd administration**	−1.05 ± 2.29
	**28 days post** **3rd administration**	−1.26 ± 2.45
	**3 months post** **3rd administration**	−1.79 ± 2.61

SD—Standard Deviation; ALS-FRSr—Amyotrophic Lateral Sclerosis Functional Rating Scale revised.

**Table 2:** Correlation analysis between selected factors using Spearman’s correlation rank test.

	Age	Disease Duration	CSF C4 I 0d	CSF C3b II 7d	CSF C4 III 0d	CRP I 0d	CRP III 0d	Neu II 0d	Neu III 0d
Age					0.99 **				
Plasma C3b I 0d		0.58 *				0.70 *			
Plasma C4 I 0d			0.45 *			0.54 *			
Plasma C3b II 0d								0.82 *	
Plasma C3b II 7d				0.79 ***					
Plasma C1q III 0d									–0.99 **
Plasma C3b III 0d	–0.99 *						0.59 *		
Plasma C4 III 0d					0.54 *				
CSF C1q I 0d						0.58 *			
CSF C1q III 0d							0.73 *		

I, II, III—cell administration procedure; d—day. Level of significance—* *p* < 0.05, ** *p* < 0.005, *** *p* < 0.0001.

## Data Availability

Data available upon request from B.M.
